# Functionalized Sugarcane Bagasse for U(VI) Adsorption from Acid and Alkaline Conditions

**DOI:** 10.1038/s41598-017-18698-9

**Published:** 2018-01-15

**Authors:** Shouzheng Su, Qi Liu, Jingyuan Liu, Hongsen Zhang, Rumin Li, Xiaoyan Jing, Jun Wang

**Affiliations:** 1 Key Laboratory of Superlight Material and Surface Technology, Ministry of Education, Harbin Engineering University, Harbin, 150001 P. R. China; 2 Institute of Advanced Marine Materials, Harbin Engineering University, Harbin, 150001 P. R. China; 3 Harbin Shipbuilding Engineering Design & Research Academy, Harbin, China; 4 Modern Analysis, Test and Research Center, Heilongjiang University of Science and Technology, Harbin, 150027 P. R. China

## Abstract

The highly efficient removal of uranium from mine tailings effluent, radioactive wastewater and enrichment from seawater is of great significance for the development of nuclear industry. In this work, we prepared an efficient U(VI) adsorbent by EDTA modified sugarcane bagasse (MESB) with a simple process. The prepared adsorbent preserves high adsorptive capacity for UO_2_^2+^ (pH 3.0) and uranyl complexes, such as UO_2_(OH)^+^, (UO_2_)_2_(OH)_2_^2+^ and (UO_2_)_3_(OH)_5_^+^ (pH 4.0 and pH 5.0) and good repeatability in acidic environment. The maximum adsorption capacity for U(VI) at pH 3.0, 4.0 and 5.0 is 578.0, 925.9 and 1394.1 mg/g and the adsorption capacity loss is only 7% after five cycles. With the pH from 3.0 to 5.0, the inhibitive effects of Na^+^ and K^+^ decreased but increased of Mg^2+^ and Ca^2+^. MESB also exhibits good adsorption for [UO_2_(CO_3_)_3_]^4−^ at pH 8.3 from 10 mg/L to 3.3 μg/L. Moreover, MESB could effectively extract U(VI) from simulated seawater in the presence of other metals ions. This work provided a general and efficient uranyl enriched material for nuclear industry.

## Introduction

Uranium is a vital commodity for nuclear energy, using as fuel for electricity generation. However, mine tailings effluent and wastewater from nuclear industry are seriously harmful for ecological and human health due to its radiochemical and toxicological^1,2^. Moreover, extraction of uranium from unconventional resource such as seawater, which is nearly 1000 times higher than that available in terrestrial ores^3,4^, has received recent attention. Hence, the highly efficient enrichment of uranium from mine tailings effluent, radioactive wastewater and seawater is of great significance for the development of nuclear industry. Among all kinds of extraction methods, such as adsorption^1,5–7^, ion exchange^8–10^, chemical reduction^11,12^, biological processes^13,14^ and membrane processes^15–17^, adsorption has been widely employed to enrich uranyl form aqueous due to its low cost, simple design, and ease of operation. Recently, various kinds of organic adsorbents (such as such as amidoxime^18–20^, amine^21–23^, carboxylates^24–26^ functionalized adsorbents) and inorganic adsorbents (such as mesoporous Mg(OH)_2_^27–29^, zero-valent iron^30,31^ and other functionalized inorganic adsorbents^32,33^) have been developed.

However, few adsorbent could be applied in all of mine tailings effluent, radioactive wastewater and seawater. The extraction is limited by some reasons: (1) aqueous uranium species is various, such as cationic UO_2_^2+^, UO_2_OH^+^ and (UO_2_)_2_(OH)^2+^ in mine tailings effluent and radioactive wastewater^34,35^ and anionic [UO_2_(CO_3_)_3_]^4−^ in seawater^36,37^, (2) the concentration of uranium is in a wide range concentrations (mg/L-μg/L), (3) there are large amount of competing metal ions, such as sodium, potassium, magnesium and calcium, and (4) adsorbent should have a great chemical and structural stability in both acidic (mine tailings effluent and radioactive wastewater) and alkaline (seawater) environment. Therefore, an adsorbent with remarkable adsorption properties and stability is necessary.

Agricultural waste materials are of particular interest since they are produced in great amounts and available worldwide^38–43^. These materials represent an interesting and attractive alternative as biosorbents because of their physico-chemical characteristic, particular structure, chemical stability, and high reactivity resulting from the presence of abundance functional groups on the surface. Moreover, it is well known that agricultural waste materials are renewable and biodegradable. In the application of adsorption, the excellent performance of different agricultural waste materials has been demonstrated, such as crop straws^44^, sawdust^45–47^, rice bran^48,49^, corncobs^50,51^, etc, which all have been widely studied for removing heavy metals from aqueous solution. Sugarcane bagasse (SB), a complex material containing cellulose, hemicellulose and lignin as major constituents, with high stability, abundant hydroxyl groups and low cost, has proven to be good candidate for supporting other functional compounds^52,53^. Recently, many efforts have been devoted to the preparation of SB-based composites for their applications in metal ion removal. For example, Yu *et al*. investigated the separation of Cu^2+^ and Pb^2+^ by tetraethylenepentamine modified SB^54^; Ramos *et al*. grafted phthalate on SB for the uptake of Co^2+^, Cu^2+^, and Ni^2+^ ^55^; Palin *et al*. evaluated the biosorption of Pb^2+^ utilizing SB colonized by Basidiomycetes^56^. Therefore, it would be attractive to introduce SB as the substrate to fabricate adsorbent for uranium extraction.

EDTA is a kind of biodegradable chelating agent which could form stable (1:1) complexes with rare earth ions in aqueous solution^57,58^. In this study, EDTA was introduced to SB through esterification for adsorption U(VI) from aqueous solutions and the composites (MESB) could be easily recycled by magnetic separation avoiding secondary pollution and loss by magnetic functionalization. This work aimed to investigate whether the adsorbent could efficiently extract uranium from mine tailings effluent, radioactive wastewater and seawater, including: (1) test the adsorption properties for uranium from acidic (pH = 3.0, 4.0 and 5.0) and alkaline (pH = 8.3) conditions, (2) evaluate the stability by regeneration efficiency and reusability, (3) determine the effects of co-existing cations ions (K^+^, Na^+^ Mg^2+^ and Ca^2+^) and (4) examined the adsorption for low-concentration U(VI) (μg/L) with a vast amount of other metals ions. This work provides both fundamental knowledge and practical aspects that are valuable for interpreting adsorption of uranium on MESB and for effective enrichment of uranium from mine tailings effluent, radioactive wastewater and seawater.

## Results and Discussion

**Figure 1:**
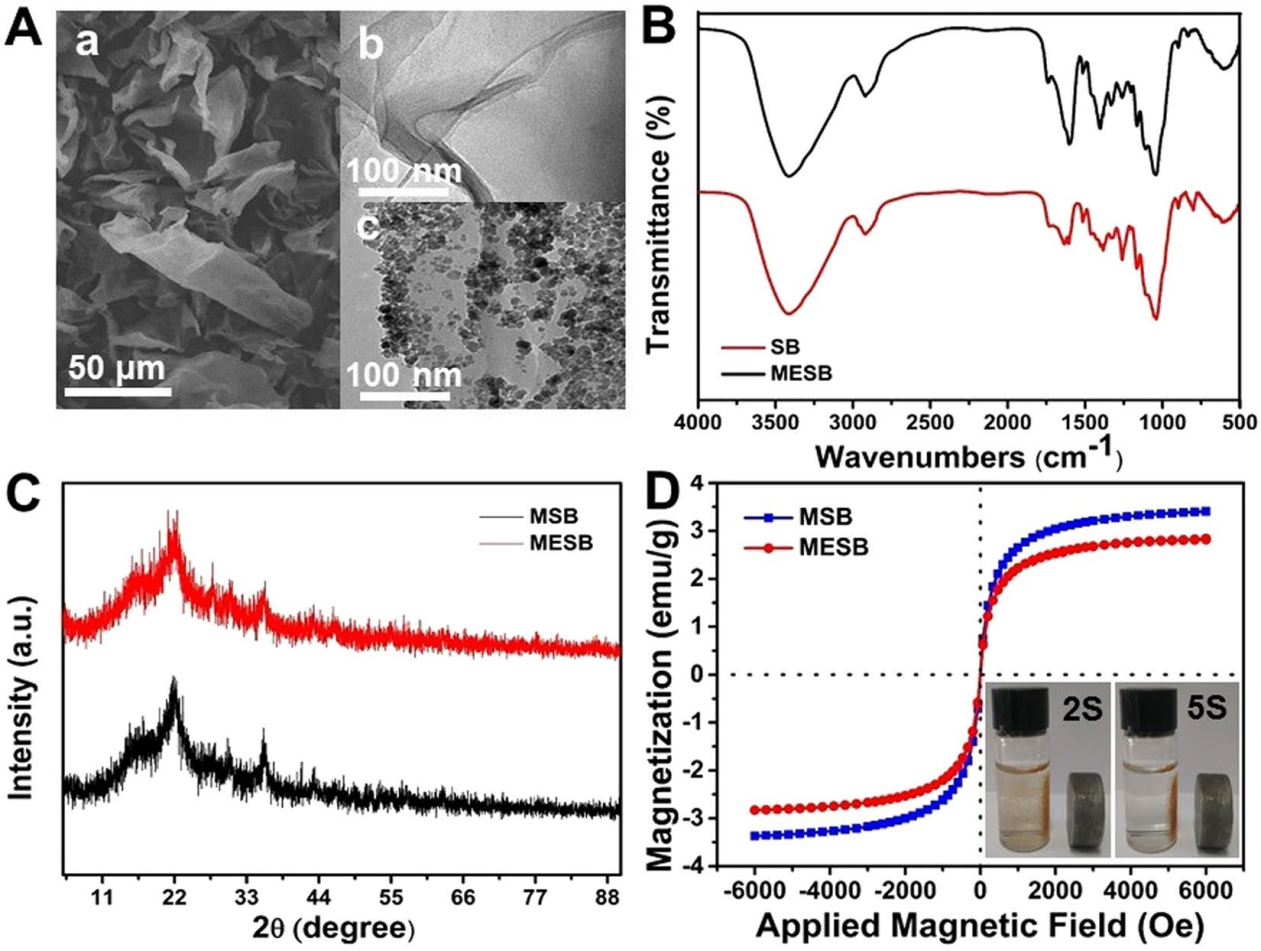
SEM of MESB (**A**a) and TEM of SB (**A**b) and MESB (**A**c). FTIR spectra of SB and MESB (**B**). XRD pattern of MSB and MESB (**C**), and Magnetization curve of MSB and MESB.

The morphology and structure of SB and MESB before and after the adsorption of U(VI) were characterized by SEM and TEM measurements. As shown in Fig. 1Aa, MESB is present as wrinkled and sheet-like structure. The SEM image for SB is shown in Fig. 1Ab. It can be seen that the pristine SB shows a sheet-like structure with smooth surface and tiny wrinkles. After co-precipitation to form MSB composite, Fe_3_O_4_ NPs are decorated on the surface of SB. TEM image (Figure S1) clearly shows that SB are the matrix of the Fe_3_O_4_ NPs, and the average size of the Fe_3_O_4_ NPs is about 10 nm. As shown in Fig. 1Ac, the magnetic NPs are not broken and maintained regular shapes, during the introduction of EDTA. After the adsorption of U(VI), the sheet-like structure of SB and MESB is retained (Figure S2A and B). Figure S2C shows U(VI) exist on the surface of SB in the form of small particles. Because only hydroxyl groups of SB could interact with U(VI), a small amount of uranium is adsorbed. However, the coverage of U(VI) on the surface of MESB increases significantly after modified by EDTA in Figure S2D.

Figure 1B gives the FT-IR spectra for SB and MSBE. As can be seen, the comparative FTIR spectra of both materials show intense band at 3400 cm^−1^ and 1051 cm^−1^ assigned to the vibrational modes of hydroxyl groups present in the polysaccharide fraction of SB. The absorption bands at 2902 cm^−1^ should be assigned to the stretching vibration of C-H. In the FT-IR spectrum of SB, the bands at 1636 and 1608 cm^−1^ are correspond to the stretching vibration of hemicellulose of C=O and the stretch vibration of C-O, respectively. Compared with the spectrum of SB, in the spectrum of MESB, the appearance of strong bands at 1749 cm^−1^ can be attributed to axial deformation of the ester bond, and the arising of strong bands at 1599 and 1396 cm^−1^ are correspond to the asymmetric and symmetric stretching of the carboxylate ion, respectively. The ester and acid IR bands indicate that EDTAD acylated the hydroxy group of SB to generate an ester bond with consequent release of a carboxylic acid functional group.

X-ray diffraction patterns of MSB and MSBE are shown in Fig. 1C. The intense peaks of MSB at 2θ values of 30.1°, 35.5°, 43.3°, 53.4°, 57.2°, and 62.5° indexed to (220) (311) (400) (422) (511) and (440), respectively, which are consistent with the standard XRD data, indicating that the Fe_3_O_4_ NPs were successfully coated on SB. The intense peaks slightly reduced after introducing EDTA.

The magnetic properties were investigated with a VSM. According to Fig. 1D, the VSM plot showed a value of 3.41 emu/g for the saturation magnetization of MSB and no hysteresis loop in the magnetization^59^. This magnetic susceptibility value is reasonable to believe that the simple and rapid separation of MSB can be achieved under a magnetic field. For MESB, a saturation magnetization of 2.82 emu/g was obtained, while decreased to 0.59 emu/g due to the shielding of EDTA coating resulting from the modification process. However, the MESB with declined saturation magnetization value also possesses enough magnetic response to meet the need of magnetic separation, which would bring a great convenience in the practical applications of the adsorbent (the inset of Fig. 1D).

Table 1 shows the elemental analysis results of SB, MSB and MSBE. It can be seen that the nitrogen content decreased drastically when MSB was obtained. This may be attributed to the decomposition of N-containing organic of SB under basic conditions. While, the nitrogen contents were increased from 0.40 to 1.55 after esterification. The obvious increase of nitrogen content also can be observed from the decrease of mass ratio of carbon and nitrogen from 104.98 to 25.08, which could prove the introduction of the EDTA. Moreover, it was possible to determinate the concentration of EDTA moiety in the modified materials (*C*_ED TA_ (0.411 mmol/g)).

**Table 1:** Elemental analysis results of SB, MSB, and MESB.

Compound	Elemental content %			
	C	N	H	C/N
SB	42.82	0.70	6.53	—
MSB	41.99	0.40	5.86	104.98
MESB	38.87	1.55	5.73	25.08

### U(VI) adsorption from acidic conditions

**Figure 2:**
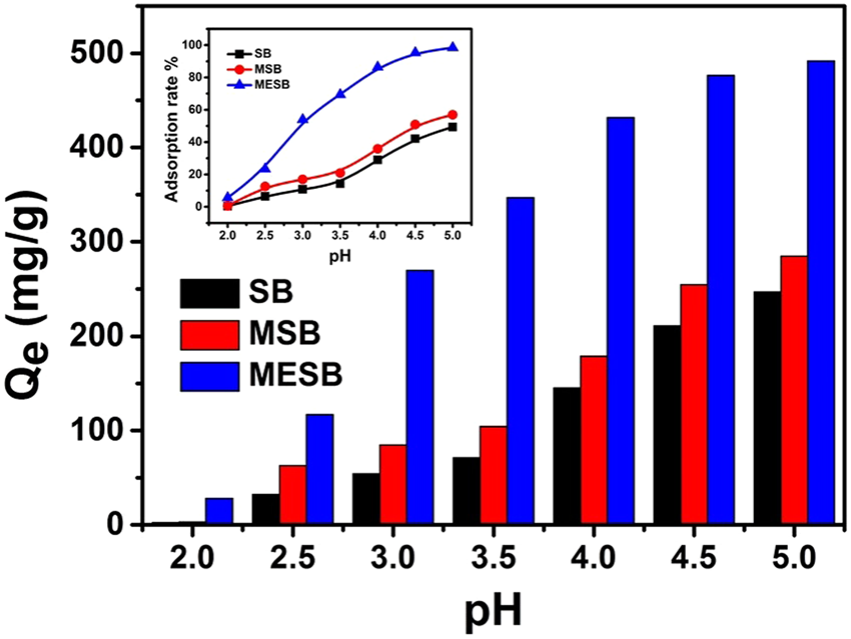
Effect of pH on U(VI) adsorption on MESB and and adsorption rate (insert). (C_0_ = 100 mg/L, V = 50 mL, T = 298 K, m = 0.02 g, t = 60 min).

The complexation of heavy metal ions by a chelating ligand strongly depends on pH, because the pH of the adsorption medium affect the presence form of metal ions greatly and also have a strongly influence on the adsorbents especially the ones containing functional groups^60,61^. U(VI) from mine tailings effluent and radioactive wastewater common exists in acid conditions, which limited adsorption to sorbents for positively charged and electrostatically repel the positive uranyl species. Moreover, most of the adsorbents are difficult to be stable under acidic conditions and the environment will be caused secondary pollution. As shown in Fig. 2, the effect of pH on the adsorption capacity of U(VI) by SB, MSB and MESB was evaluated in a pH range of 2.0–5.0. It is clear that MESB is more helpful to adsorb U(VI) in acid conditions than SB and MSB. The pH_zpc_ (pH zero point charge, Figure S3) of MESB is found to be 4.0. When pH < 4.0, carboxyl groups are completely protonated. Although repulsive electrostatic interactions with H^+^, the adsorption rate of positively charged uranyl ion is still maintained above 50%. As the pH values increased from 4.0 to 5.0, the surface charge of MESB is negative, which is prone to adsorb positively charged metal ions on their surfaces and the adsorption rate reaches 86.3% and 98.3% (the inset of Fig. 2).

**Figure 3:**
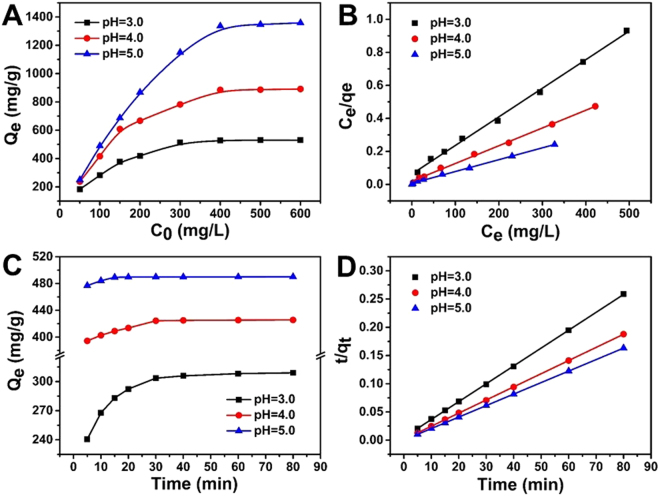
Effect of initial concentration of MESB (**A**) and corresponding Langmuir adsorption isotherms (**B**) at pH 3.0, 4.0 and 5.0 (A). (V = 50 mL, T = 298 K, m = 0.02 g, t = 60 min) Effect of contact time of MESB (**C**) and their corresponding pseudo second-order kinetics (**D**). (C_0_ = 100 mg/L, V = 50 mL, T = 298 K, m = 0.02 g).

Figure 3A shows the equilibrium adsorption capacity of U(VI) on MESB as a function of different initial uranium concentrations at pH 3.0, 4.0 and 5.0. The adsorption capacity increases rapidly with the initial concentration increasing until equilibrium and the equilibrium adsorption capacity at pH 3.0, 4.0 and 5.0 are 530.15, 981.25 mg/g and 1358.75, respectively.

The adsorption isotherms reveal the interactive behaviors between the adsorbent and adsorbate, which have been simulated by utilizing the well-established fundamental models. Langmuir and Freundlich isotherm models expressed in Eqs. (1) and (2), respectively, are used to understand the adsorption mechanism in this paper.

1 $$\frac{{{\rm{C}}}_{{\rm{e}}}}{{{\rm{Q}}}_{{\rm{e}}}}=\frac{{\rm{Ce}}}{{{\rm{Q}}}_{{\rm{\max }}}}+\frac{1}{{{\rm{K}}}_{{\rm{L}}}{{\rm{Q}}}_{{\rm{\max }}}}$$

2 $${{\rm{logQ}}}_{{\rm{e}}}={{\rm{logK}}}_{{\rm{F}}}+\frac{1}{{\rm{n}}}{{\rm{logc}}}_{{\rm{e}}}$$

where C_e_ is the concentration of the adsorbate in solution at equilibrium (mg/L), Q_e_ is the amount of U(VI) adsorbed at equilibrium (mg/L), and Q_max_ represents the saturated monolayer adsorption capacity (mg/g) of Langmuir isotherm model^62^. K_L_ is the Langmuir adsorption constant related to the energy of adsorption (L/mg). K_F_ is the Freundlich constant related to the adsorption capacity [(mg/g) (L/mg)^1/n^] and n is the Freundlich exponent related to adsorption intensity. The Freundlich model is suitable for the nonideal adsorption on the heterogeneous surfaces as well as multilayer adsorption^63^.

Langmuir and Freundlich isotherm parameters calculated from fitting processes are listed in Table S1 and shown in Fig. 3B. It can be seen that the Langmuir equation fits the experimental data better than the Freundlich model with a higher correlation coefficient (R^2^) at pH 3.0, 4.0 and 5.0, implying that the adsorption process results in the formation of a monolayer coverage of various uranium species on MESB. The maximum adsorption capacity of MESB was evaluated as 578.0, 925.9 and 1394.1 mg/g at pH 3.0, 4.0 and 5.0, respectively. Previous studies have demonstrated that the major component of uranium is UO_2_^2+^ at pH 3.0, and then become complicated, such as UO_2_(OH)^+^, (UO_2_)_2_(OH)_2_^2+^ and (UO_2_)_3_(OH)_5_^+^ with pH increased to 5.0^64^. The results demonstrate that MESB is favorable for the adsorption of various uranium species, which is probably due to the strong chelating ability of EDTA.

Furthermore, the essential characteristics of the Langmuir isotherm can be described by a separation factor, R_L_^65^, which is defined as follows:

3 $${{\rm{R}}}_{{\rm{L}}}=\frac{1}{1+{{\rm{K}}}_{{\rm{L}}}{{\rm{c}}}_{0}}$$

where K_L_ is the Langmuir equilibrium constant and C_0_ is the initial concentration of metal ion. The value of R_L_ provides guidance for the possibility of the adsorption process to proceed. R_L_ > 1.0, unsuitable; R_L_ = 1.0, linear; 0 < R_L_ < 1.0, suitable; R_L_ = 0, irreversible. The values of R_L_, were found to range from 0.4200 to 0.0569, 0.2606 to 0.0285 and 0.1492 to 0.0144 at pH 3.0, 4.0 and 5.0, respectively, indicating the suitability of MESB as adsorbents for the adsorption of U(VI) ions at acid conditions (Figure S4).

Another factor can help understanding the behavior of the adsorption of U(VI) ions on MESB, is the Langmuir surface coverage rate (θ), which relates the surface coverage of the fiber to the initial concentration of U(VI) ions^66^ and can be calculated using the following equation:

4 $${\rm{\theta }}=\frac{{{\rm{K}}}_{{\rm{L}}}{{\rm{c}}}_{0}}{1+{{\rm{K}}}_{{\rm{L}}}{{\rm{c}}}_{0}}$$

The relationship of θ and initial concentration of U(VI) ions was depicted in Figure S5. Evidently, the adsorption of U(VI) ions on MESB in the early age was very fast (low coverage of fiber surface and plenty of free active sites are available for binding with the metal ions) then tends to be a plateau at higher surface coverage where most of the active sites are occupied. This implies the applicability of Langmuir model to describe the adsorption of U(VI) ions on MESB.

The effect of contact time was investigated in a kinetics study of the adsorption process. Figure 3C shows the time profile of U(VI) adsorption at pH 3.0, 4.0 and 5.0 in terms of adsorption capacity. It is observed that adsorption generally achieved the equilibrium within 60 min. The results indicate that higher pH not only has higher adsorption rate but also higher adsorption efficiency.

The following pseudo-first-order^67^ and pseudo-second-order^68^ models are employed to further interpret the kinetic data:

5 $$\mathrm{Pseudo} \mbox{-} \mathrm{first} \mbox{-} \mathrm{order}\,{\rm{model}}:\,\mathrm{ln}({{\rm{q}}}_{{\rm{e}}}-{{\rm{q}}}_{{\rm{t}}})={{\rm{k}}}_{1}{\rm{t}}+{{\rm{lnq}}}_{{\rm{e}}}$$

6 $$\mathrm{Pseudo} \mbox{-} \mathrm{second} \mbox{-} \mathrm{order}\,{\rm{model}}:\frac{{\rm{t}}}{{{\rm{q}}}_{{\rm{t}}}}=\frac{{\rm{t}}}{{{\rm{k}}}_{2}{{{\rm{q}}}_{{\rm{e}}}}^{2}}+\frac{{\rm{t}}}{{{\rm{q}}}_{{\rm{e}}}}$$

where q_e_ and q_t_ (mg/g) are the adsorption capacities at equilibrium and at time t (min), respectively; k_1_ and k_2_ are the rate constant of the pseudo-first-order and pseudo-second-order model. The corresponding kinetic parameters from both models are listed in Table S2. Higher correlation coefficient (R^2^ > 0.99 for both) as well as value of q_e,cal_ (276.2, 438.6,and 490.2 mg/g) at pH 3.0, 4.0 and 5.0, approximating to q_e,exp_ (273.7, 433.7, and 490.1 mg/g), indicates that pseudo-second-order model describes the adsorption process better (Fig. 3D). This model is based on the assumption that the rate-limiting step of the reaction is due to chemical adsorption^69^.

In order to investigate if pore or film diffusion was the controlling step in the adsorption, intra-particle diffusion model was further tested as follows^70^:

7 $${{\rm{Q}}}_{{\rm{t}}}={{\rm{kt}}}^{0.5}+{\rm{C}}$$

Figure S6 shows the intra-particle diffusion plots for the U(VI) adsorption on MESB at pH 3.0, 4.0 and 5.0. It is apparent that two linear portion appear in all of the plots, which indicates that the adsorption is affected by two steps. This could be explained as follows: the first portion represents instantaneous adsorption or external surface adsorption stage, in which large numbers of U(VI) are adsorbed rapidly by COO- of MESB. After almost all the exterior COO- is occupied, the second portion of the plots is nearly parallel, suggesting that equilibrium state reached at last. The intra-particle diffusion rate constants in every step follow the order of k_pH5_ > k_pH4_ > k_pH3_, which are presented in Table S2. At low pH abundant of hydronium ion (H_3_O^+^) could compete with U(VI) for binding on the functional groups (binding sites). Moreover, when pH < 4.0 the surface charge of MESB is positive, which is difficult to adsorb positively charged metal ions on its surfaces. All of these could affect the adsorption of U(VI). Therefore, the adsorption has a shorter equilibrium time at pH 5.0 than 4.0 and 3.0.

**Figure 4:**
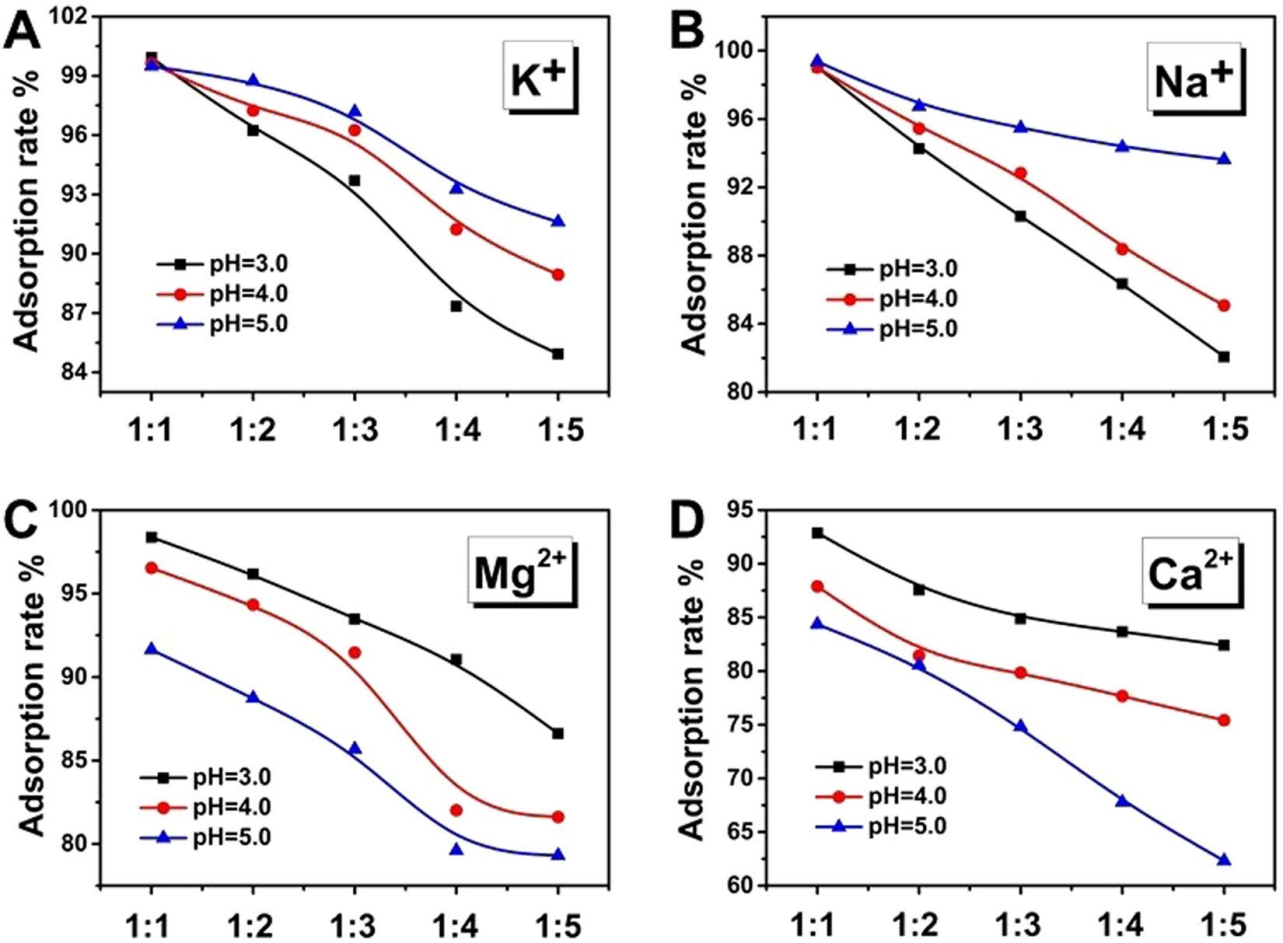
Effect of coexisting ions on U(VI) adsorption on MESB at pH 3.0, 4.0 and 5.0. (C_0_ = 100 mg/L, V = 50 mL, T = 298 K, m = 0.02 g, t = 60 min).

Table S3 shows effects of coexisting K^+^ on U(VI) adsorption by MESB at pH = 3.0, 4.0 and 5.0. It was observed that the adsorption of U(VI) decreased with the increase of K^+^ concentration at any pH (Fig. 4A). However, as the pH increased from 3.0 to 5.0, the inhibitive effects decreased. The effect of Na^+^ on the adsorption of U(VI) is the same as that of K^+^ (Fig. 4B). K^+^ and Na^+^ cannot coordinate with EDTA but can be adsorbed onto the surface of MESB by electrostatic interactions. At low pH, a large number of H^+^ concentrated on negatively charged surface of MESB and then the surface turns positive resulting in repulsion for cations. Na^+^ and K^+^ are easier to aggregate onto surface of adsorbents due to their smaller ionic radius than uranium (0.102, 0.138 and 0.604–0.684 nm for Na^+^, K^+^ and UO_2_^2+^)^71^, which make adsorbents more positive leading to decrease of adsorption towards uranyl. Therefore, effect on the adsorption of uranium is stronger with the increase in concentration of Na^+^ and K^+^. With the increase of pH, the electrostatic repulsion of adsorbent and ion decreases gradually with decrease in contents of H^+^ in solutions, which makes the coordination between adsorbents and uranyl strengthened so as to increase the adsorption rate. In Fig. 4C and D, the adsorption rate of uranium decreases with the increasing concentration of Mg^2+^ and Ca^2+^ the same as that of Na^+^ and K^+^. However, with the increase of pH, the effect of coexisting Mg^2+^ and Ca^2+^ was opposite with Na^+^ and K^+^. Based on previous studies, EDTA can be coordinated with Mg^2+^ and Ca^2+^ and higher pH (acidic condition) is favor for coordination. As a result, high concentration of Mg^2+^ and Ca^2+^ and high pH (pH = 5) will have a greater impact on the adsorption of uranium. Overall, the inhibitive effects of coexisting ions follow the order of: Ca^2+^ > Mg^2+^ > Na^+^ > K^+^ and affected by pH.

**Figure 5:**
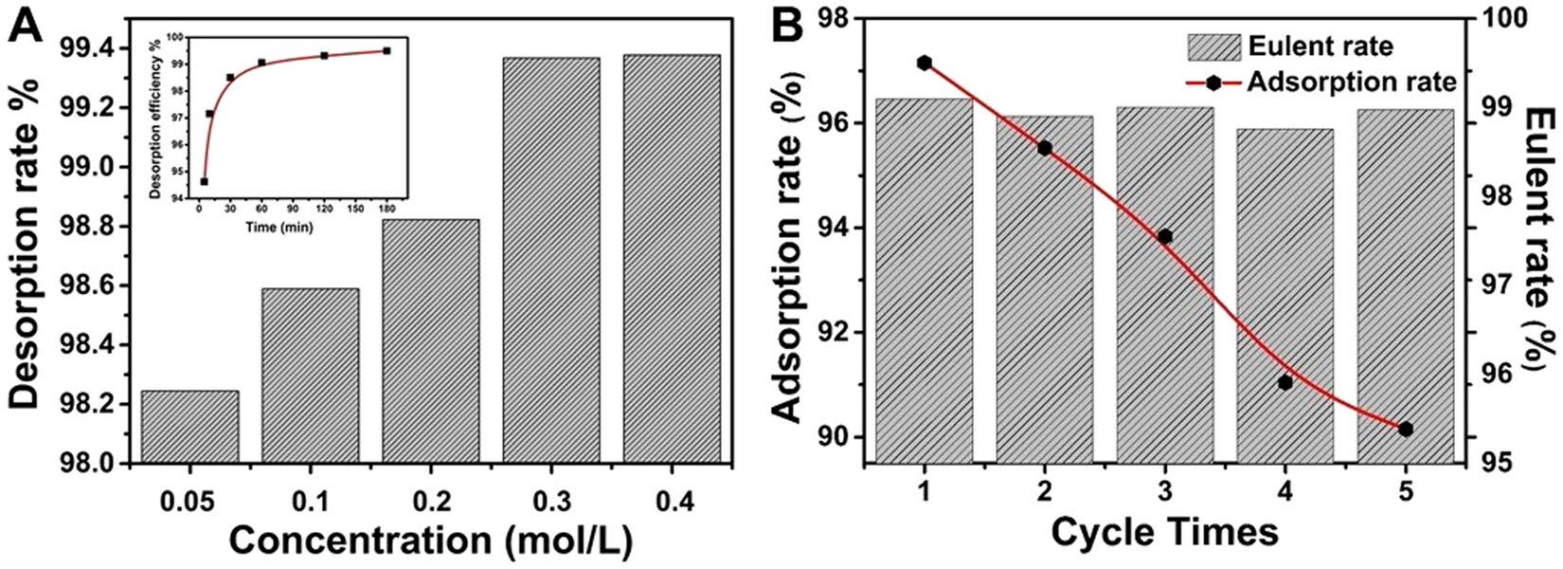
Desorption rate of MESB at different concentrations of HNO_3_ (**A**) and effect of time on U(VI) desorption (insert). Recycling of the MESB in the adsorption of U(VI) (**B**).

The reusability and stability of MESB are of great importance for practical application. According to the previous result, MESB have poor U(VI) adsorption ability when pH < 2. HNO_3_ (0.05–0.4 mol/L) is chosen to evaluate the reutilization performance. The results obtained are given in Fig. 5A. The elution efficiency is above 99% when the acid concentration is greater than 0.3 mol/L, suggesting that HNO_3_ is an effective eluent for the recovery of the adsorbent. And 99% of U(VI) will be desorbed within an hour (the inset of Fig. 5A). Therefore, reusability experiments are subsequently estimated using 0.3 mol/L HNO_3_ as the desorbing agent and the results are shown Fig. 5B. It is observed that regenerated MESB still has high adsorption capacity even after 5 cycles of adsorption/desorption, indicating that MESB is a stable and recyclable adsorbent. After desorption, the eluent is first adjusted to neutral and then add NH_4_OH to get (NH_4_)_2_U_2_O_7_ precipitate, which can be further reduced to UO_2_ by H_2_:

$${{\rm{2UO}}}_{2}{({{\rm{NO}}}_{3})}_{2}+6{{\rm{NH}}}_{4}{\rm{OH}}\to {({{\rm{NH}}}_{4})}_{2}{{\rm{U}}}_{2}{{\rm{O}}}_{7}+4{{\rm{NH}}}_{4}{{\rm{NO}}}_{3}+2{{\rm{H}}}_{2}{\rm{O}}$$

$${({{\rm{NH}}}_{4})}_{2}{{\rm{U}}}_{2}{{\rm{O}}}_{7}+{{\rm{H}}}_{2}\to {{\rm{UO}}}_{2}+2{{\rm{NH}}}_{3}+3{{\rm{H}}}_{2}{\rm{O}}$$

Therefore, the acceptable adsorption ability and regeneration ability proved that MESB has a potential application prospect for the preconcentration of U(VI) from acidic conditions.

### U(VI) adsorption from alkaline conditions

Although estimated 4.5 billion tons of uranium dissolved in seawater which is much larger than the terrestrial sources, the concentration of uranium is extremely low at ∼3.3 μg/L and is found to be present principally as the anionic triscarbonatouranato (VI) [UO_2_(CO_3_)_3_]^4−^ species. Moreover, basic pH (~8.3), and myriad of other metals present also contribute to the challenge of oceanic extractions.

In order to determine the adsorption property of [UO_2_(CO_3_)_3_]^4−^ on MESB, the adsorption process was carried on 10 mg/L of [UO_2_(CO_3_)_3_]^4−^ at pH 8.3. According to Figure S7, most uptake of U(VI) occurred in the first 60 min, and full adsorption equilibrium was reached within 120 min. At equilibrium, the U(VI) adsorption capacities was 100 mg/g with an adsorption efficiency of 73.2%. The result indicates MESB also has excellent adsorption ability for [UO_2_(CO_3_)_3_]^4−^ at alkaline conditions. A higher correlation coefficient (R^2^) and a value of q_e_,_cal_ (151.7 mg/g) that is close to q_e_,_exp_ (150.2 mg/g) indicates that the adsorption process follows the pseudo-second-order model.

**Figure 6:**
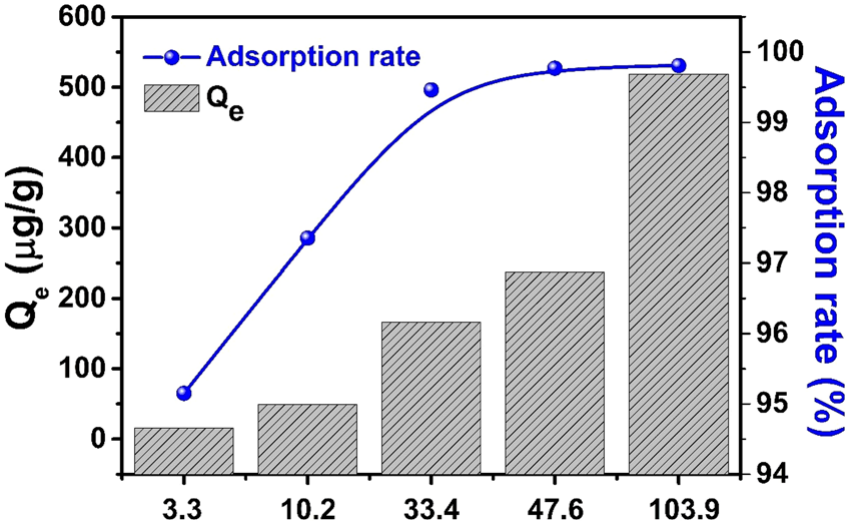
The adsorption rate of U\(VI) by MESB in simulated seawater. (V = 50 mL, T = 298 K, m = 0.02 g, t = 12 h).

To further investigate whether MESB is suitable for the extraction of uranium from seawater, we examined the applicability of adsorbent in the extraction of simulated seawater with low concentration of U(VI) and a vast amount of other metals ions with similar or even higher concentrations (Table S4). As shown in Fig. 6, U(VI) is still effectively adsorbed onto MESB not only at initial concentration of 100 ppb but also at an extremely low concentration of 3.3 ppb. These results suggest that MESB is a promising uranium adsorbent even for seawater where the basic pH, low concentration and competition of coexisting cations to U(VI) is overwhelming.

## Conclusions

We have reported here a general and efficient uranyl enriched material, EDTA modified sugarcane bagasse, for nuclear industry. The prepared adsorbent preserved good adsorption properties in both acidic (mine tailings effluent and radioactive wastewater) and alkaline (seawater) environment. Under acid conditions, MESB was favorable for the adsorption of UO_2_^2+^ (pH 3.0) and uranyl complexes, such as UO_2_(OH)^+^, (UO_2_)_2_(OH)_2_^2+^ and (UO_2_)_3_(OH)_5_^+^ (pH 4.0 and pH 5.0) and the adsorption capacity was 578.0, 925.9 and 1394.1 mg/g at pH 3.0, pH 4.0 and pH 5.0, respectively. The coexisting of Na^+^, K^+^, Mg^2+^ and Ca^2+^ could decrease the adsorption capacity and affected by pH. With the pH from 3.0 to 5.0, the inhibitive effected of Na^+^ and K^+^ decreased but increased of Mg^2+^ and Ca^2+^. MESB also exhibited excellent chemical and structural stability in acid eluent and the adsorption capacity loss was only 7% after five cycles. Under alkaline conditions, MESB show strong coordination to [UO_2_(CO_3_)_3_]^4−^ at pH 8.3. Moreover, MESB could effectively extract low concentration U(VI) (103.9–3.3 μg/L) from simulated seawater in the presence of other metals ions. This work provided a general and efficient uranyl enriched material for nuclear industry.

## Experimental Section

### Materials

SB was repeatedly washed with deionized water to remove dirt and soluble impurities and then dried at 80 °C for 24 h. Subsequently, they were crushed and sieved to a particle size of under 150 (0.1 mm) meshes using a standard sieve. Ethylenediamine tetraacetic acid (EDTA) was purchased from Xiya (China). N,N-dimethylformamide (DMF) was purchased from Fuyu (China). Other reagents obtained from Kermel, China, were analytical grade. All the chemical reagents in this work were used without any further treatment.

### Preparation of magnetic SB

Magnetic SB (MSB) was prepared by a coprecipitation method. In a 150 mL beaker, 0.417 g of ferrous chloride tetrahydrate (FeCl_2_·4H_2_O) and 0.405 g of ferric chloride hexahydrate (FeCl_3_·6H_2_O) were taken and dissolved by the addition of 45 mL water containing 3 g SB under ultrasound irradiation. Then, ammonium hydroxide (NH_3_·H_2_O) solution was added dropwise into the beaker until the pH reached 10. Black precipitates appeared immediately after adding ammonium hydroxide solution. During the reaction process, the temperature was maintained at 60 °C. The magnetic adsorbent was collected by magnetic separation after 1 h reaction and washed several times with water and ethanol. Finally, MSB was obtained after dried at 60 °C for 3 h.

### Preparation of MESB

EDTAD preparation method was described by Capretta *et al*.^72^. The prepared EDTAD (1.0 g) was added to 100 mL of N,N-dimethylformamide (DMF) containing 1.0 g of MSB in a three neck round bottom flask. After the mixture was stirred at 60 °C for 4 h, MESB was separated by an external magnet and washed with DMF, saturated sodium bicarbonate solution and deionized water successively. Then MESB was dried at 60 °C for 6 h and preserved in a desiccator.

### Characterization

MESB was characterized by several techniques. The morphology of the material were examined by scanning electron microscope (SEM, JSM-6480A), transmission electron microscopy (TEM, FEI Tecnai G2 S-Twin). X-ray diffraction (XRD) analysis was performed on a Rigaku D/max-IIIB diffractometer with Cu Kα irradiation (λ = 1.54178 Å) for determining the structure. The diffractgrams were recorded in the 2θ range 5**°**–90**°**. Qualitative chemical structure assessment was done by FT-IR analysis (PerkinElmer Spectrum 100) in a range of 4000–500 cm^−1^. A vibrating sample magnetometer (VSM, LakeShore 7304) was used to characterize the magnetic properties. The element content was measured by element analyzer (Vario Macro). The concentrations of metal ions were analyzed using inductively coupled plasma-atomic emission spectroscopy (ICP-AES, Optima-7000DV).

### Adsorption tests

Individual stock solution of 1000 mg/L U(VI) was prepared by dissolving UO_2_(NO_3_)_2_·6H_2_O in deionized water. Adsorption experiments were performed in a 50 mL U(VI) solution with initial concentrations of 50, 100, 150, 200, 300, 400, 500, or 600 mg/L, 10 mg adsorbent and the solution pH adjusted to 3.0, 4.0 and 5.0 with HNO_3_ or NaOH. The effect of solution pH was evaluated in the case of 100 mg/L U(VI), 10 mg adsorbent and where the contact time was 60 min. For kinetics studies, the solution was shaken at 200 rpm for 5, 10, 15, 20, 30, 40, 60 or 80 min. Uranyl carbonate was prepared following the methodology described by Sapretta *et al*.^73^ and adjusted pH to 8.3.

### Competing of coexisting ions tests

The effect of coexisting ions for uranium (VI) adsorption onto MESB was tested through a 1-h adsorption process at 298 K. The solution used in this experiment contained four common monovalent and bivalent cations-Na^+^, K^+^, Mg^2+^ and Ca^2+^, with the concentration 1~5 times than uranium (VI) (mole ratio). An enhanced simulated seawater test was performed following our previous work^74^. MESB was added into simulated seawater containing U(VI) with initial concentrations of 3, 10, 30, 50 or 100 μg/L, after shaking at 200 rpm for 120 min, the residual U(VI) ions in solution was analysed by ICP-MS.

### Regeneration and recyclability tests

After adsorption experiments, MESB was washed thoroughly with deionized water until U(VI) ion was not detected in the rinsing solution and then dried in a vacuum oven at room temperature. 50 mL of 0.05–0.4 mol/L HNO_3_ solution was mixed with the dried MESB for desorption. After the mixture was shaken for 5, 10, 30, 60, 120 or 180 min, the concentrations of U(VI) in the solution were analyzed. The experiment was repeated five times in order to evaluate the recyclability of MESB.

## Electronic supplementary material

Supplementary information
