# Supplementary material for: Functionalized Sugarcane Bagasse for U(VI) Adsorption from Acid and Alkaline Conditions
Source: Sci Rep. 2018 Jan 15;8:793. doi: 10.1038/s41598-017-18698-9 (PMC5768879; doi:10.1038/s41598-017-18698-9)
Supplement: Supplementary file 1 — Supplementary information [file 41598_2017_18698_MOESM1_ESM.doc]

*Electronic Supplementary Information for*

**Functionalized Sugarcane Bagasse for U(VI) Adsorption from Acid and Alkaline Conditions**

Shouzheng Su,^1^ Qi Liu,^2,3^ * Jingyuan Liu,^1^ Hongsen Zhang,^4^ Rumin Li,^1^ Xiaoyan Jing,^1^ Jun Wang ^1, 2, 3^ *


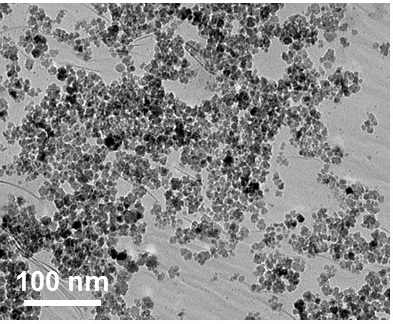


Figure S1. TEM of MSB.


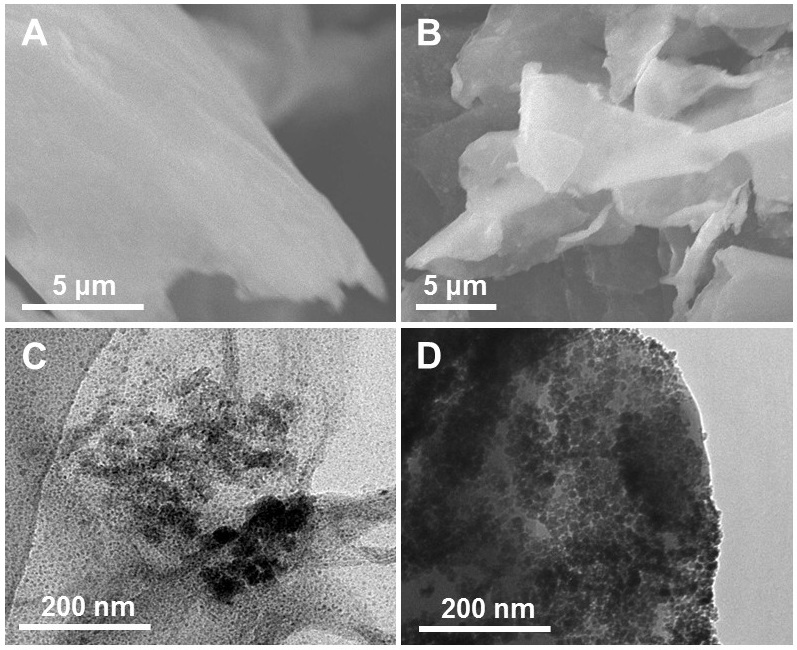


Figure S2. SEM and TEM images of SB (A, C) and MESB (B, D).


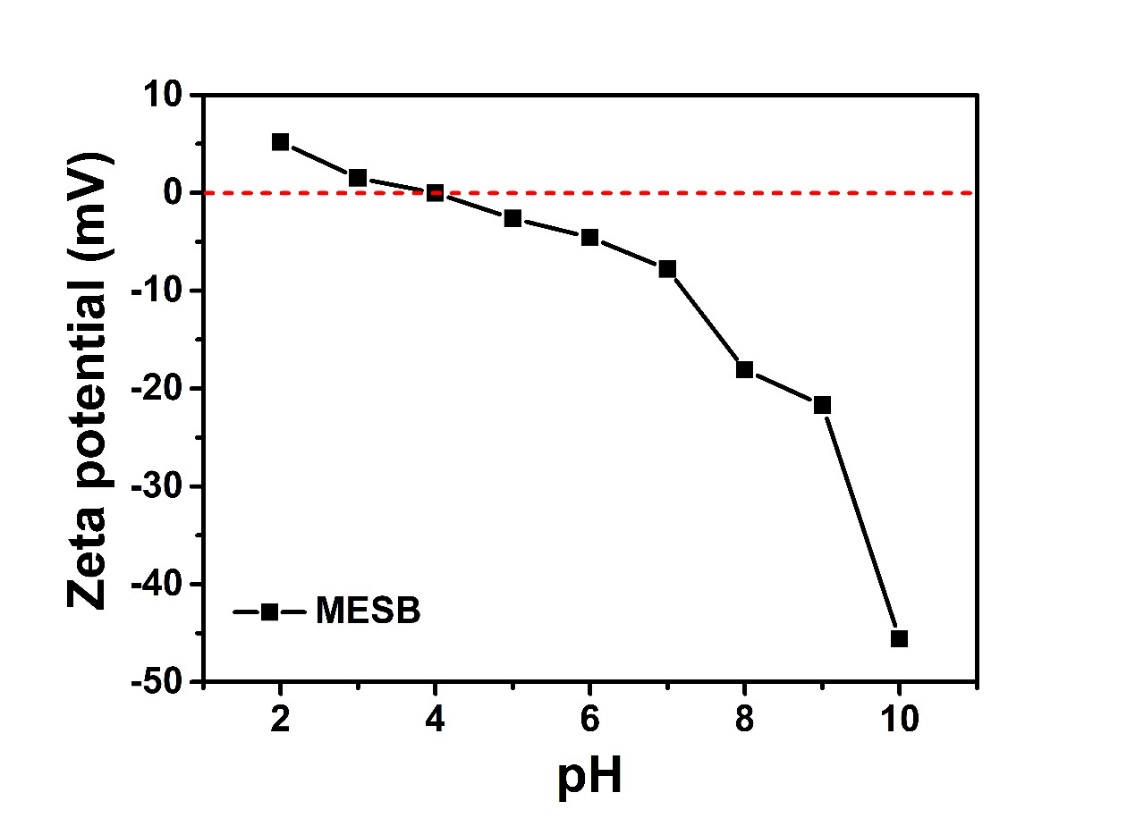


Figure S3. Zeta potential of MESB.


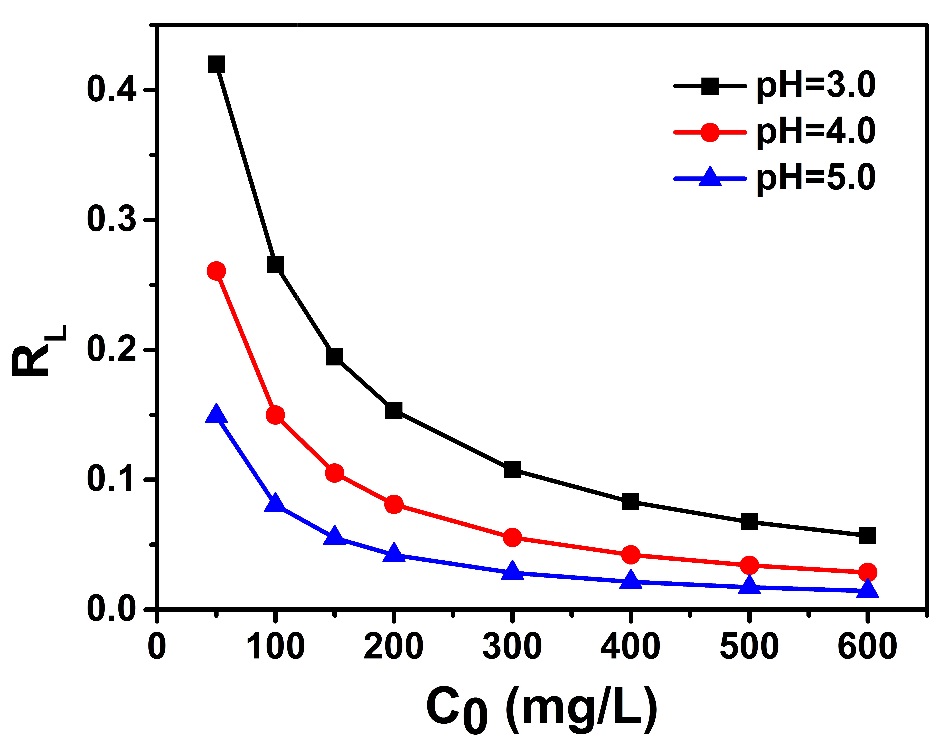


Figure S4. Langmuir separation factor (R_L_) plots.


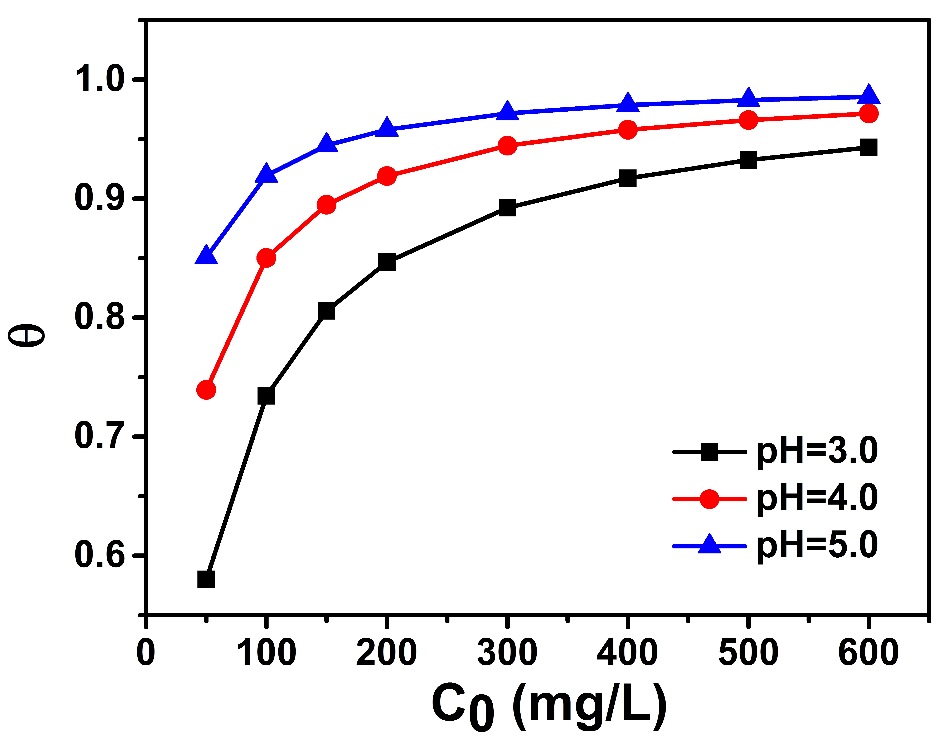


Figure S5. Langmuir surface coverage (θ) plots.


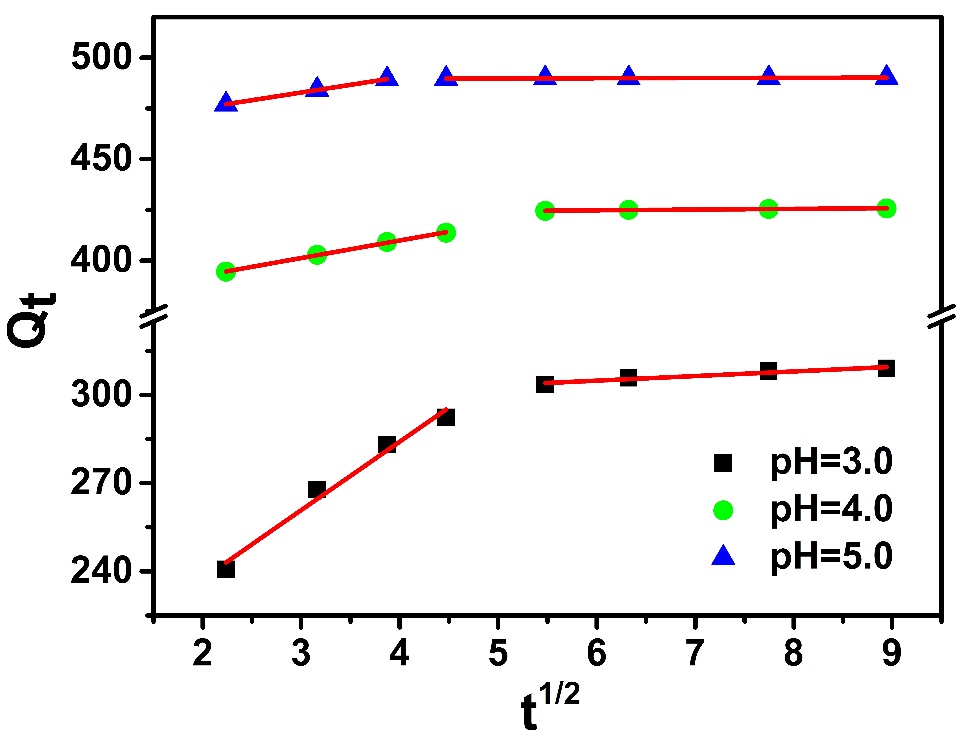


Figure S6. The intra-particle diffusion model fitted for the adsorption of U(VI).


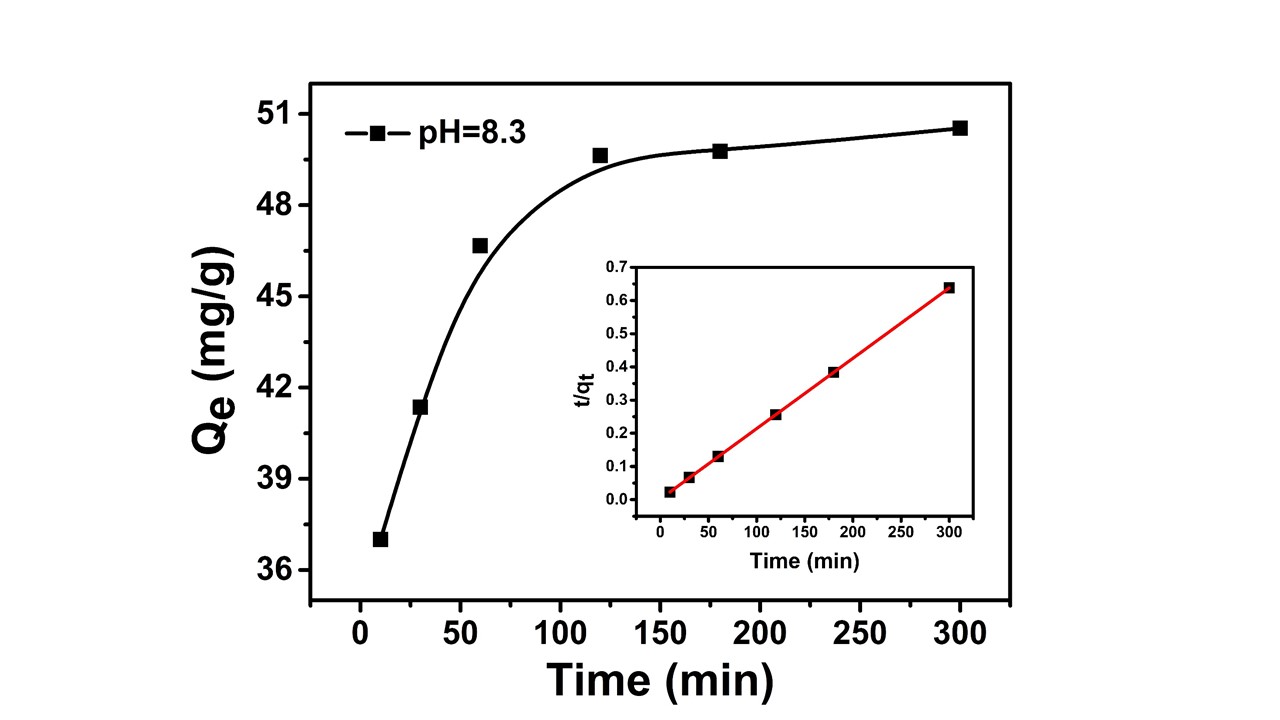


Figure S7. Effect of contact time on U(VI) adsorption on MESB at pH 8.3 and pseudo-second-order model (insert). (V = 50 mL, T = 298 K, m = 0.02 g, t=300 min)

**Table S1**

Isotherm parameter for adsorption of U (VI) on MESB

| pH | Langmuir isotherm | | | Freundlich isotherm | | |
| --- | --- | --- | --- | --- | --- | --- |
|  | Q_max_ (mg/g) | K_L_(L/mg) | R^2^ | K_F_(L/mg) | n | R^2^ |
| 3.0 | 578.0 | 0.0276 | 0.9973 | 91.62 | 3.287 | 0.9391 |
| 4.0 | 925.9 | 0.0567 | 0.9982 | 208.2 | 3.834 | 0.9710 |
| 5.0 | 1394.1 | 0.1140 | 0.9975 | 504.8 | 5.731 | 0.9646 |

**Table S2**

Kinetic parameter for adsorption of U(VI) on MESB

| Kinetics model | pH | K | q_e,exp_(mg/g) | q_e,cal_(mg/g) | R^2^ |
| --- | --- | --- | --- | --- | --- |
| Pseudo-first  order | 3.0 | 6.727*10^-2^ | 273.7 | 233.7 | 0.9243 |
|  | 4.0 | 7.115*10^-2^ | 433.7 | 361.7 | 0.9791 |
|  | 5.0 | 5.255*10^-2^ | 490.2 | 485.8 | 0.6618 |
| Pseudo-second order | 3.0 | 9.340*10^-4^ | 273.7 | 276.2 | 0.9998 |
|  | 4.0 | 3.889*10^-3^ | 433.7 | 438.6 | 0.9999 |
|  | 5.0 | 7.117*10^-3^ | 490.1 | 490.2 | 0.9999 |
| Intra-particle diffusion | 3.0 | 23.28/1.565 | 273.7 | 309.5 | 0.9991/0.7882 |
|  | 4.0 | 8.664/0.3489 | 433.7 | 425.7 | 0.9986/0.9229 |
|  | 5.0 | 7.575/0.1024 | 490.1 | 490.2 | 0.9733/0.9136 |

**Table S3**

Isotherm parameter for adsorption of U (VI) on MESB

|  | Adsorption rate of U(VI) (%) | | | | | | | | | | | |
| --- | --- | --- | --- | --- | --- | --- | --- | --- | --- | --- | --- | --- |
|  | pH=3.0 | | | | pH=4.0 | | | | pH=5.0 | | | |
|  | K | Na | Mg | Ca | K | Na | Mg | Ca | K | Na | Mg | Ca |
| 1:1 | 99.9 | 99.0 | 98.3 | 92.8 | 99.6 | 99.0 | 96.5 | 87.8 | 99.4 | 99.3 | 91.6 | 84.3 |
| 1:2 | 96.2 | 94.2 | 96.1 | 87.5 | 97.2 | 95.4 | 94.3 | 81.4 | 98.7 | 96.7 | 88.7 | 80.5 |
| 1:3 | 93.6 | 90.3 | 93.4 | 84.8 | 96.2 | 92.8 | 91.4 | 79.8 | 97.1 | 95.4 | 85.6 | 74.8 |
| 1:4 | 87.3 | 86.3 | 91.0 | 83.6 | 91.2 | 88.3 | 82.0 | 77.6 | 93.2 | 94.3 | 79.6 | 67.7 |
| 1:5 | 84.9 | 82.0 | 86.6 | 82.4 | 88.9 | 85.0 | 81.6 | 75.4 | 91.6 | 93.6 | 79.3 | 62.3 |

**Table S4**

The extraction of U(VI) by MESB in simulated seawater.

| Initial concentration (μg/L) | 3.3 | 10.2 | 33.4 | 47.6 | 103.9 |
| --- | --- | --- | --- | --- | --- |
| Residual concentration (μg/L) | 0.16 | 0.27 | 0.18 | 0.11 | 0.2 |
| Adsorption rate (%) | 95.2 | 97.4 | 99.5 | 99.8 | 99.8 |
